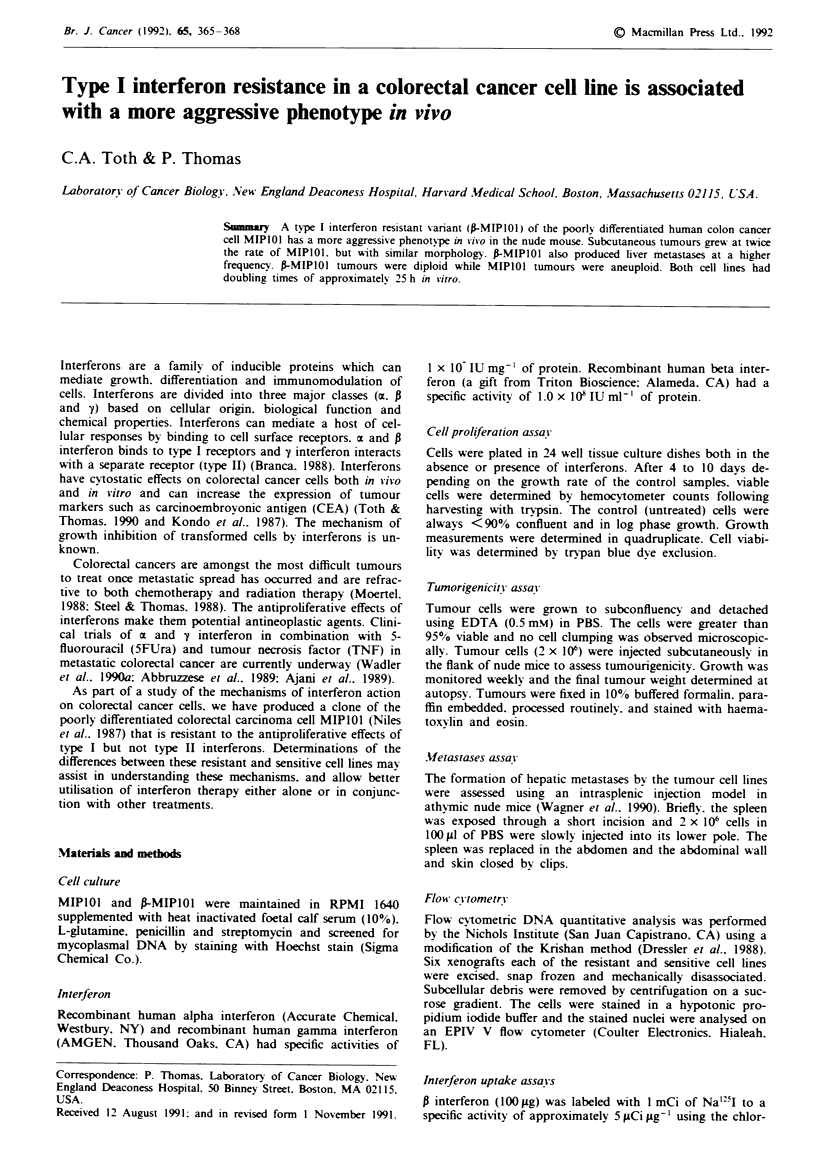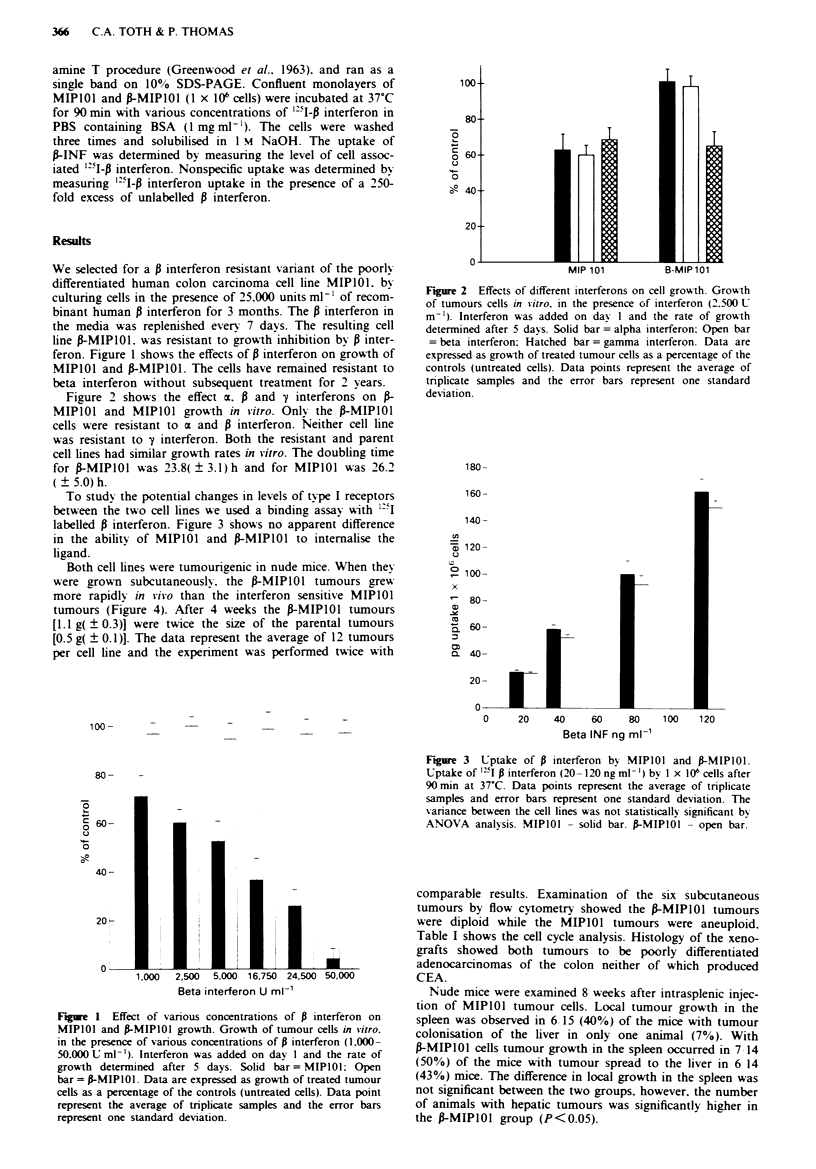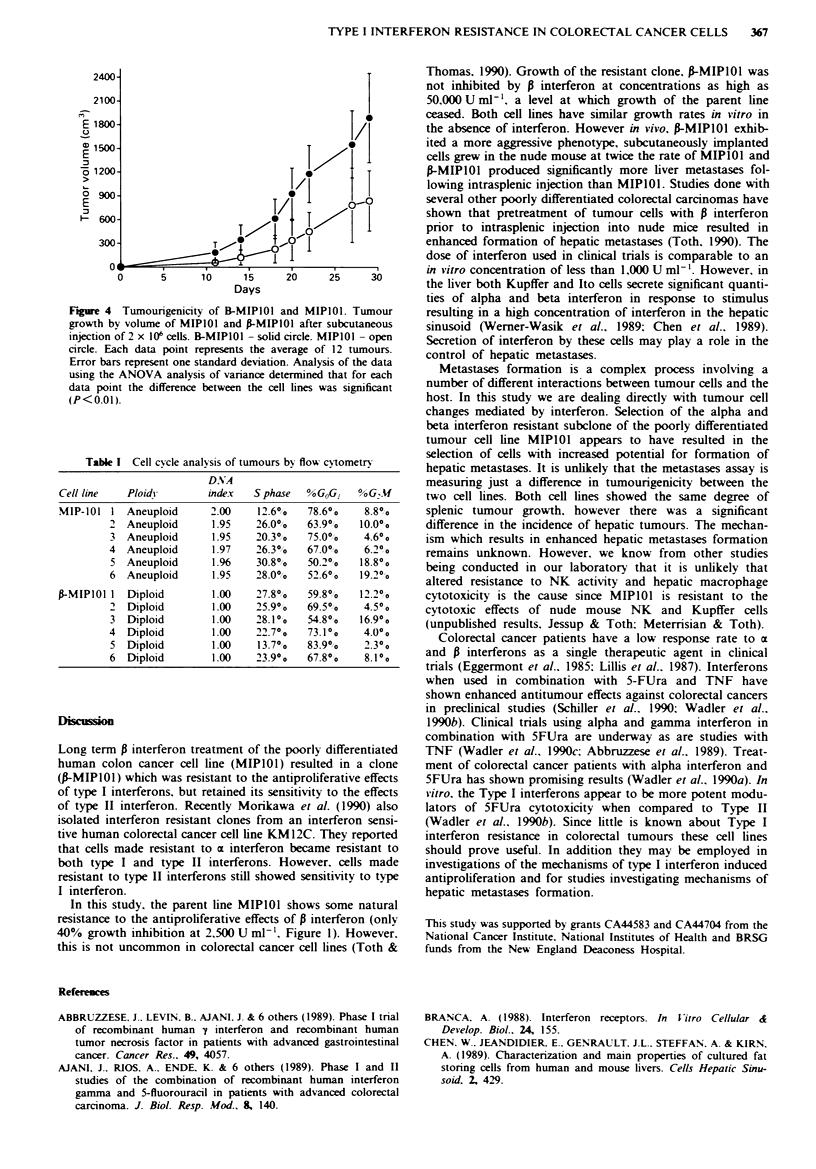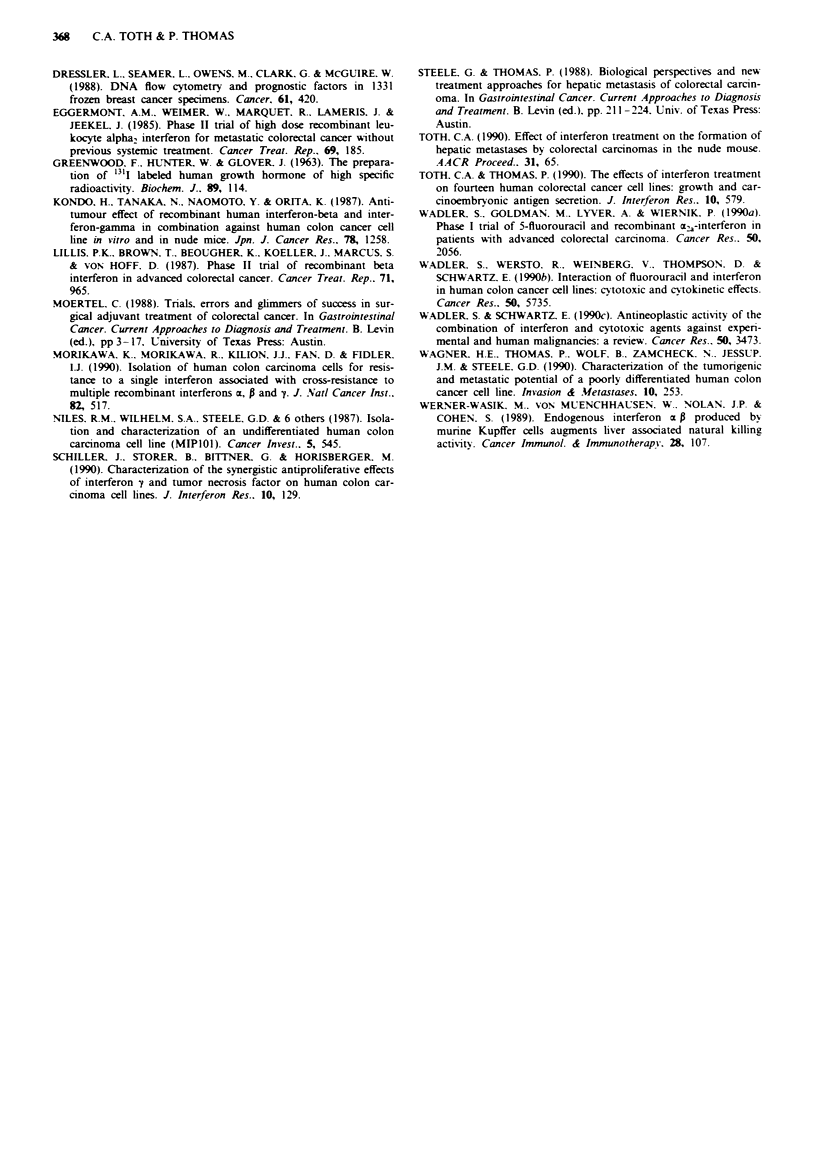# Type I interferon resistance in a colorectal cancer cell line is associated with a more aggressive phenotype in vivo.

**DOI:** 10.1038/bjc.1992.74

**Published:** 1992-03

**Authors:** C. A. Toth, P. Thomas

**Affiliations:** Laboratory of Cancer Biology, New England Deaconess Hospital, Harvard Medical School, Boston, Massachusetts 02115.

## Abstract

A type I interferon resistant variant (beta-MIP101) of the poorly differentiated human colon cancer cell MIP101 has a more aggressive phenotype in vivo in the nude mouse. Subcutaneous tumours grew at twice the rate of MIP101, but with similar morphology. beta-MIP101 also produced liver metastases at a higher frequency. beta-MIP101 tumours were diploid while MIP101 tumours were aneuploid. Both cell lines had doubling times of approximately 25 h in vitro.


*Br. J. Cancer* (1992), **65**, 365-368 © Macmillan Press Ltd., 1992

### Type I interferon resistance in a colorectal cancer cell line is associated with a more aggressive phenotype *in vivo*

C.A. Toth & P. Thomas

*Laboratory of Cancer Biology, New England Deaconess Hospital, Harvard Medical School, Boston, Massachusetts 02115, USA.*

**Summary** A type I interferon resistant variant (β-MIP101) of the poorly differentiated human colon cancer cell MIP101 has a more aggressive phenotype *in vivo* in the nude mouse. Subcutaneous tumours grew at twice the rate of MIP101, but with similar morphology. β-MIP101 also produced liver metastases at a higher frequency. β-MIP101 tumours were diploid while MIP101 tumours were aneuploid. Both cell lines had doubling times of approximately 25 h *in vitro*.

Interferons are a family of inducible proteins which can mediate growth, differentiation and immunomodulation of cells. Interferons are divided into three major classes (α, β and γ) based on cellular origin, biological function and chemical properties. Interferons can mediate a host of cellular responses by binding to cell surface receptors, α and β interferon binds to type I receptors and γ interferon interacts with a separate receptor (type II) (Branca, 1988). Interferons have cytostatic effects on colorectal cancer cells both *in vivo* and *in vitro* and can increase the expression of tumour markers such as carcinoembroyonic antigen (CEA) (Toth & Thomas, 1990 and Kondo *et al*., 1987). The mechanism of growth inhibition of transformed cells by interferons is unknown.

Colorectal cancers are amongst the most difficult tumours to treat once metastatic spread has occurred and are refractive to both chemotherapy and radiation therapy (Moertel, 1988; Steel & Thomas, 1988). The antiproliferative effects of interferons make them potential antineoplastic agents. Clinical trials of α and γ interferon in combination with 5-fluorouracil (5FUra) and tumour necrosis factor (TNF) in metastatic colorectal cancer are currently underway (Wadler *et al*., 1990*a*; Abbruzzese *et al*., 1989; Ajani *et al*., 1989).

As part of a study of the mechanisms of interferon action on colorectal cancer cells, we have produced a clone of the poorly differentiated colorectal carcinoma cell MIP101 (Niles *et al*., 1987) that is resistant to the antiproliferative effects of type I but not type II interferons. Determinations of the differences between these resistant and sensitive cell lines may assist in understanding these mechanisms, and allow better utilisation of interferon therapy either alone or in conjunction with other treatments.

### Materials and methods

### Cell culture

MIP101 and β-MIP101 were maintained in RPMI 1640 supplemented with heat inactivated foetal calf serum (10%), L-glutamine, penicillin and streptomycin and screened for mycoplasmal DNA by staining with Hoechst stain (Sigma Chemical Co.).

### Interferon

Recombinant human alpha interferon (Accurate Chemical, Westbury, NY) and recombinant human gamma interferon (AMGEN, Thousand Oaks, CA) had specific activities of $1 \times 10^7$ IU mg$^{-1}$ of protein. Recombinant human beta interferon (a gift from Triton Bioscience; Alameda, CA) had a specific activity of $1.0 \times 10^8$ IU ml$^{-1}$ of protein.

### Cell proliferation assay

Cells were plated in 24 well tissue culture dishes both in the absence or presence of interferons. After 4 to 10 days depending on the growth rate of the control samples, viable cells were determined by hemocytometer counts following harvesting with trypsin. The control (untreated) cells were always <90% confluent and in log phase growth. Growth measurements were determined in quadruplicate. Cell viability was determined by trypan blue dye exclusion.

### Tumorigenicity assay

Tumour cells were grown to subconfluency and detached using EDTA (0.5 mM) in PBS. The cells were greater than 95% viable and no cell clumping was observed microscopically. Tumour cells ($2 \times 10^6$) were injected subcutaneously in the flank of nude mice to assess tumourigenicity. Growth was monitored weekly and the final tumour weight determined at autopsy. Tumours were fixed in 10% buffered formalin, paraffin embedded, processed routinely, and stained with haematoxylin and eosin.

### Metastases assay

The formation of hepatic metastases by the tumour cell lines were assessed using an intrasplenic injection model in athymic nude mice (Wagner *et al*., 1990). Briefly, the spleen was exposed through a short incision and $2 \times 10^6$ cells in 100 μl of PBS were slowly injected into its lower pole. The spleen was replaced in the abdomen and the abdominal wall and skin closed by clips.

### Flow cytometry

Flow cytometric DNA quantitative analysis was performed by the Nichols Institute (San Juan Capistrano, CA) using a modification of the Krishan method (Dressler *et al*., 1988). Six xenografts each of the resistant and sensitive cell lines were excised, snap frozen and mechanically disassociated. Subcellular debris were removed by centrifugation on a sucrose gradient. The cells were stained in a hypotonic propidium iodide buffer and the stained nuclei were analysed on an EPIV V flow cytometer (Coulter Electronics, Hialeah, FL).

### Interferon uptake assays

β interferon (100 μg) was labeled with 1 mCi of $Na^{125}I$ to a specific activity of approximately 5 μCi μg$^{-1}$ using the chlor-

Correspondence: P. Thomas, Laboratory of Cancer Biology, New England Deaconess Hospital, 50 Binney Street, Boston, MA 02115, USA.
Received 12 August 1991; and in revised form 1 November 1991.

amine T procedure (Greenwood *et al.*, 1963), and ran as a single band on 10% SDS-PAGE. Confluent monolayers of MIP101 and β-MIP101 ($1 \times 10^6$ cells) were incubated at 37°C for 90 min with various concentrations of $^{125}$I-β interferon in PBS containing BSA (1 mg ml$^{-1}$). The cells were washed three times and solubilised in 1 M NaOH. The uptake of β-INF was determined by measuring the level of cell associated $^{125}$I-β interferon. Nonspecific uptake was determined by measuring $^{125}$I-β interferon uptake in the presence of a 250-fold excess of unlabelled β interferon.

### Results

We selected for a β interferon resistant variant of the poorly differentiated human colon carcinoma cell line MIP101, by culturing cells in the presence of 25,000 units ml$^{-1}$ of recombinant human β interferon for 3 months. The β interferon in the media was replenished every 7 days. The resulting cell line β-MIP101, was resistant to growth inhibition by β interferon. Figure 1 shows the effects of β interferon on growth of MIP101 and β-MIP101. The cells have remained resistant to beta interferon without subsequent treatment for 2 years.

Figure 2 shows the effect α, β and γ interferons on β-MIP101 and MIP101 growth *in vitro*. Only the β-MIP101 cells were resistant to α and β interferon. Neither cell line was resistant to γ interferon. Both the resistant and parent cell lines had similar growth rates *in vitro*. The doubling time for β-MIP101 was 23.8(±3.1) h and for MIP101 was 26.2 (±5.0) h.

To study the potential changes in levels of type I receptors between the two cell lines we used a binding assay with $^{125}$I labelled β interferon. Figure 3 shows no apparent difference in the ability of MIP101 and β-MIP101 to internalise the ligand.

Both cell lines were tumourigenic in nude mice. When they were grown subcutaneously, the β-MIP101 tumours grew more rapidly *in vivo* than the interferon sensitive MIP101 tumours (Figure 4). After 4 weeks the β-MIP101 tumours [1.1 g(±0.3)] were twice the size of the parental tumours [0.5 g(±0.1)]. The data represent the average of 12 tumours per cell line and the experiment was performed twice with comparable results. Examination of the six subcutaneous tumours by flow cytometry showed the β-MIP101 tumours were diploid while the MIP101 tumours were aneuploid. Table I shows the cell cycle analysis. Histology of the xenografts showed both tumours to be poorly differentiated adenocarcinomas of the colon neither of which produced CEA.

Nude mice were examined 8 weeks after intrasplenic injection of MIP101 tumour cells. Local tumour growth in the spleen was observed in 6/15 (40%) of the mice with tumour colonisation of the liver in only one animal (7%). With β-MIP101 cells tumour growth in the spleen occurred in 7/14 (50%) of the mice with tumour spread to the liver in 6/14 (43%) mice. The difference in local growth in the spleen was not significant between the two groups, however, the number of animals with hepatic tumours was significantly higher in the β-MIP101 group ($P<0.05$).

**Figure 1** Effect of various concentrations of β interferon on MIP101 and β-MIP101 growth. Growth of tumour cells *in vitro*, in the presence of various concentrations of β interferon (1,000–50,000 U ml$^{-1}$). Interferon was added on day 1 and the rate of growth determined after 5 days. Solid bar = MIP101; Open bar = β-MIP101. Data are expressed as growth of treated tumour cells as a percentage of the controls (untreated cells). Data point represent the average of triplicate samples and the error bars represent one standard deviation.

**Figure 2** Effects of different interferons on cell growth. Growth of tumours cells *in vitro*, in the presence of interferon (2,500 U m$^{-1}$). Interferon was added on day 1 and the rate of growth determined after 5 days. Solid bar = alpha interferon; Open bar = beta interferon; Hatched bar = gamma interferon. Data are expressed as growth of treated tumour cells as a percentage of the controls (untreated cells). Data points represent the average of triplicate samples and the error bars represent one standard deviation.

**Figure 3** Uptake of β interferon by MIP101 and β-MIP101. Uptake of $^{125}$I β interferon (20–120 ng ml$^{-1}$) by $1 \times 10^6$ cells after 90 min at 37°C. Data points represent the average of triplicate samples and error bars represent one standard deviation. The variance between the cell lines was not statistically significant by ANOVA analysis. MIP101 – solid bar. β-MIP101 – open bar.

**Figure 4** Tumourigenicity of B-MIP101 and MIP101. Tumour growth by volume of MIP101 and β-MIP101 after subcutaneous injection of 2 × $10^6$ cells. B-MIP101 - solid circle. MIP101 - open circle. Each data point represents the average of 12 tumours. Error bars represent one standard deviation. Analysis of the data using the ANOVA analysis of variance determined that for each data point the difference between the cell lines was significant ($P < 0.01$).

**Table I** Cell cycle analysis of tumours by flow cytometry

| *Cell line* | | *Ploidy* | *DNA index* | *S phase* | *%$G_0G_1$* | *%$G_2M$* |
|---|---|---|---|---|---|---|
| MIP-101 | 1 | Aneuploid | 2.00 | 12.6% | 78.6% | 8.8% |
| | 2 | Aneuploid | 1.95 | 26.0% | 63.9% | 10.0% |
| | 3 | Aneuploid | 1.95 | 20.3% | 75.0% | 4.6% |
| | 4 | Aneuploid | 1.97 | 26.3% | 67.0% | 6.2% |
| | 5 | Aneuploid | 1.96 | 30.8% | 50.2% | 18.8% |
| | 6 | Aneuploid | 1.95 | 28.0% | 52.6% | 19.2% |
| β-MIP101 | 1 | Diploid | 1.00 | 27.8% | 59.8% | 12.2% |
| | 2 | Diploid | 1.00 | 25.9% | 69.5% | 4.5% |
| | 3 | Diploid | 1.00 | 28.1% | 54.8% | 16.9% |
| | 4 | Diploid | 1.00 | 22.7% | 73.1% | 4.0% |
| | 5 | Diploid | 1.00 | 13.7% | 83.9% | 2.3% |
| | 6 | Diploid | 1.00 | 23.9% | 67.8% | 8.1% |

### Discussion

Long term β interferon treatment of the poorly differentiated human colon cancer cell line (MIP101) resulted in a clone (β-MIP101) which was resistant to the antiproliferative effects of type I interferons, but retained its sensitivity to the effects of type II interferon. Recently Morikawa *et al.* (1990) also isolated interferon resistant clones from an interferon sensitive human colorectal cancer cell line KM12C. They reported that cells made resistant to α interferon became resistant to both type I and type II interferons. However, cells made resistant to type II interferons still showed sensitivity to type I interferon.

In this study, the parent line MIP101 shows some natural resistance to the antiproliferative effects of β interferon (only 40% growth inhibition at 2,500 U $ml^{-1}$, Figure 1). However, this is not uncommon in colorectal cancer cell lines (Toth & Thomas, 1990). Growth of the resistant clone, β-MIP101 was not inhibited by β interferon at concentrations as high as 50,000 U $ml^{-1}$, a level at which growth of the parent line ceased. Both cell lines have similar growth rates *in vitro* in the absence of interferon. However *in vivo*, β-MIP101 exhibited a more aggressive phenotype, subcutaneously implanted cells grew in the nude mouse at twice the rate of MIP101 and β-MIP101 produced significantly more liver metastases following intrasplenic injection than MIP101. Studies done with several other poorly differentiated colorectal carcinomas have shown that pretreatment of tumour cells with β interferon prior to intrasplenic injection into nude mice resulted in enhanced formation of hepatic metastases (Toth, 1990). The dose of interferon used in clinical trials is comparable to an *in vitro* concentration of less than 1,000 U $ml^{-1}$. However, in the liver both Kupffer and Ito cells secrete significant quantities of alpha and beta interferon in response to stimulus resulting in a high concentration of interferon in the hepatic sinusoid (Werner-Wasik *et al.*, 1989; Chen *et al.*, 1989). Secretion of interferon by these cells may play a role in the control of hepatic metastases.

Metastases formation is a complex process involving a number of different interactions between tumour cells and the host. In this study we are dealing directly with tumour cell changes mediated by interferon. Selection of the alpha and beta interferon resistant subclone of the poorly differentiated tumour cell line MIP101 appears to have resulted in the selection of cells with increased potential for formation of hepatic metastases. It is unlikely that the metastases assay is measuring just a difference in tumourigenicity between the two cell lines. Both cell lines showed the same degree of splenic tumour growth, however there was a significant difference in the incidence of hepatic tumours. The mechanism which results in enhanced hepatic metastases formation remains unknown. However, we know from other studies being conducted in our laboratory that it is unlikely that altered resistance to NK activity and hepatic macrophage cytotoxicity is the cause since MIP101 is resistant to the cytotoxic effects of nude mouse NK and Kupffer cells (unpublished results, Jessup & Toth; Meterrisian & Toth).

Colorectal cancer patients have a low response rate to α and β interferons as a single therapeutic agent in clinical trials (Eggermont *et al.*, 1985; Lillis *et al.*, 1987). Interferons when used in combination with 5-FUra and TNF have shown enhanced antitumour effects against colorectal cancers in preclinical studies (Schiller *et al.*, 1990; Wadler *et al.*, 1990*b*). Clinical trials using alpha and gamma interferon in combination with 5FUra are underway as are studies with TNF (Wadler *et al.*, 1990*c*; Abbruzzese *et al.*, 1989). Treatment of colorectal cancer patients with alpha interferon and 5FUra has shown promising results (Wadler *et al.*, 1990*a*). *In vitro*, the Type I interferons appear to be more potent modulators of 5FUra cytotoxicity when compared to Type II (Wadler *et al.*, 1990*b*). Since little is known about Type I interferon resistance in colorectal tumours these cell lines should prove useful. In addition they may be employed in investigations of the mechanisms of type I interferon induced antiproliferation and for studies investigating mechanisms of hepatic metastases formation.

This study was supported by grants CA44583 and CA44704 from the National Cancer Institute, National Institutes of Health and BRSG funds from the New England Deaconess Hospital.

### References

ABBRUZZESE, J., LEVIN, B., AJANI, J. & 6 others (1989). Phase I trial of recombinant human γ interferon and recombinant human tumor necrosis factor in patients with advanced gastrointestinal cancer. *Cancer Res.*, **49**, 4057.

AJANI, J., RIOS, A., ENDE, K. & 6 others (1989). Phase I and II studies of the combination of recombinant human interferon gamma and 5-fluorouracil in patients with advanced colorectal carcinoma. *J. Biol. Resp. Mod.*, **8**, 140.

BRANCA, A. (1988). Interferon receptors. *In Vitro Cellular & Develop. Biol.*, **24**, 155.

CHEN, W., JEANDIDIER, E., GENRAULT, J.L., STEFFAN, A. & KIRN, A. (1989). Characterization and main properties of cultured fat storing cells from human and mouse livers. *Cells Hepatic Sinusoid*, **2**, 429.

DRESSLER, L., SEAMER, L., OWENS, M., CLARK, G. & MCGUIRE, W. (1988). DNA flow cytometry and prognostic factors in 1331 frozen breast cancer specimens. *Cancer*, **61**, 420.

EGGERMONT, A.M., WEIMER, W., MARQUET, R., LAMERIS, J. & JEEKEL, J. (1985). Phase II trial of high dose recombinant leukocyte alpha$_2$ interferon for metastatic colorectal cancer without previous systemic treatment. *Cancer Treat. Rep.*, **69**, 185.

GREENWOOD, F., HUNTER, W. & GLOVER, J. (1963). The preparation of $^{131}$I labeled human growth hormone of high specific radioactivity. *Biochem. J.*, **89**, 114.

KONDO, H., TANAKA, N., NAOMOTO, Y. & ORITA, K. (1987). Antitumour effect of recombinant human interferon-beta and interferon-gamma in combination against human colon cancer cell line *in vitro* and in nude mice. *Jpn. J. Cancer Res.*, **78**, 1258.

LILLIS, P.K., BROWN, T., BEOUGHER, K., KOELLER, J., MARCUS, S. & VON HOFF, D. (1987). Phase II trial of recombinant beta interferon in advanced colorectal cancer. *Cancer Treat. Rep.*, **71**, 965.

MOERTEL, C. (1988). Trials, errors and glimmers of success in surgical adjuvant treatment of colorectal cancer. In *Gastrointestinal Cancer. Current Approaches to Diagnosis and Treatment*. B. Levin (ed.), pp 3–17, University of Texas Press: Austin.

MORIKAWA, K., MORIKAWA, R., KILION, J.J., FAN, D. & FIDLER, I.J. (1990). Isolation of human colon carcinoma cells for resistance to a single interferon associated with cross-resistance to multiple recombinant interferons α, β and γ. *J. Natl Cancer Inst.*, **82**, 517.

NILES, R.M., WILHELM, S.A., STEELE, G.D. & 6 others (1987). Isolation and characterization of an undifferentiated human colon carcinoma cell line (MIP101). *Cancer Invest.*, **5**, 545.

SCHILLER, J., STORER, B., BITTNER, G. & HORISBERGER, M. (1990). Characterization of the synergistic antiproliferative effects of interferon γ and tumor necrosis factor on human colon carcinoma cell lines. *J. Interferon Res.*, **10**, 129.

STEELE, G. & THOMAS, P. (1988). Biological perspectives and new treatment approaches for hepatic metastasis of colorectal carcinoma. In *Gastrointestinal Cancer. Current Approaches to Diagnosis and Treatment*. B. Levin (ed.), pp. 211–224, Univ. of Texas Press: Austin.

TOTH, C.A. (1990). Effect of interferon treatment on the formation of hepatic metastases by colorectal carcinomas in the nude mouse. *AACR Proceed.*, **31**, 65.

TOTH, C.A. & THOMAS, P. (1990). The effects of interferon treatment on fourteen human colorectal cancer cell lines: growth and carcinoembryonic antigen secretion. *J. Interferon Res.*, **10**, 579.

WADLER, S., GOLDMAN, M., LYVER, A. & WIERNIK, P. (1990*a*). Phase I trial of 5-fluorouracil and recombinant $\alpha_{2a}$-interferon in patients with advanced colorectal carcinoma. *Cancer Res.*, **50**, 2056.

WADLER, S., WERSTO, R., WEINBERG, V., THOMPSON, D. & SCHWARTZ, E. (1990*b*). Interaction of fluorouracil and interferon in human colon cancer cell lines: cytotoxic and cytokinetic effects. *Cancer Res.*, **50**, 5735.

WADLER, S. & SCHWARTZ, E. (1990*c*). Antineoplastic activity of the combination of interferon and cytotoxic agents against experimental and human malignancies: a review. *Cancer Res.*, **50**, 3473.

WAGNER, H.E., THOMAS, P., WOLF, B., ZAMCHECK, N., JESSUP, J.M. & STEELE, G.D. (1990). Characterization of the tumorigenic and metastatic potential of a poorly differentiated human colon cancer cell line. *Invasion & Metastases*, **10**, 253.

WERNER-WASIK, M., VON MUENCHHAUSEN, W., NOLAN, J.P. & COHEN, S. (1989). Endogenous interferon α β produced by murine Kupffer cells augments liver associated natural killing activity. *Cancer Immunol. & Immunotherapy*, **28**, 107.